# Comparative soil bacterial metabarcoding after aboveground vs. subsurface decomposition of *Mus musculus*

**DOI:** 10.1038/s41598-024-82437-0

**Published:** 2024-12-28

**Authors:** Chawki Bisker, Gillian Taylor, Helen Carney, Caroline H. Orr, Gulnaz T. Javan, Theresia Komang Ralebitso-Senior

**Affiliations:** 1 National Institute of Criminalistics and Criminology, Bouchaoui, Algiers, Algeria; 2 School of Health and Life Sciences, Teesside University, Middlesbrough, UK; 3 National Horizons Centre, Darlington, UK; 4 Department of Physical Sciences and Forensic Science Programs, Alabama State University, Montgomery, AL USA; 5 School of Pharmacy and Biomolecular Science, Liverpool John Moores University, Liverpool, UK

**Keywords:** Vertebrate decomposition, *Mus musculus*, Gravesoil metabarcoding, Postmortem interval, Clandestine grave, Microbial community succession, Metagenomics, Microbial ecology, Microbiome, Soil microbiology

## Abstract

Outdoor microcosms, metabarcoding with next-generation sequencing of the 16S rRNA bacterial gene, total body score (TBS) and physicochemical analyses were used to monitor *Mus musculus* decomposition aboveground (A) and in the subsurface (S), and compared to soil-only controls (C). As determined by MaAsLin2 analysis, significant shifts in bacterial communities at 30 cm depths within the A, S and C treatments distinguished control from experimental soils, and between aboveground and subsurface deposition, demonstrating the potential for gravesoil discrimination during the first 90 days. For example, Dokdonella (*p* = 0.0002), Edaphobaculum (*p* = 0.0004) and Lacibacter (*p* = 0.0034) recorded significant shifts relative to sampling time. Furthermore, Massilia (*p* = 0.0005), Mycobacterium (*p* = 0.0006) and Sandaracinus (*p* = 0.0007) increased in abundance for the aboveground mice treatments. This was confirmed with ANOSIM where *p* = 0.0082 showed statistically significant difference between the aboveground and subsurface deposition. TBS and physicochemical analyses suggested that nutrient release into the soils occurred during active decay and skin rapture on days 7–13 in the subsurface and days 13–20 aboveground, with a particular increase in soil potassium concentration on day 15. Significant differences in soil temperatures resulted between A and S vs. C microcosms, aligning with atmospheric temperature changes. In summary, complementary application of metabarcoding, total body score, exogenous and physicochemical methods for postmortem interval estimation and clandestine grave location highlighted the feasibility of using temperature records downloaded from meteorological stations and portable X-ray fluorescence as indicators for various phases of decomposition.

**Supplementary Information:**

The online version contains supplementary material available at 10.1038/s41598-024-82437-0.

## Introduction

Soil analysis has evolved considerably since the first mention of its potential in forensic investigation in the adventures of Sherlock Holmes^1^. Various ecological and decomposition studies explored the relationship between soil microbial community structure and diversity, the associated changes to the ecosystem, and how these infer the location of clandestine graves^2^. Despite the importance of the lower soil horizons in ecology and forensic science, these remain underexplored with most research focused on the first few centimeters of the topsoil horizon (ca. 5–10 cm). In fact, the study of the phenomena and processes associated with microbial community response to carrion decomposition in the soil substrata could be highly beneficial to forensic investigation of homicide cases outdoors.

Soil is a multiphase system, composed of organic matter, mineral material, gas, and water. It is the habitat for a vast array of organisms which, in turn, play an important role in shaping its physical and chemical properties^3^. Microbial communities form up to 95% of soil biomass and have a major effect on ecosystem functioning due to their role in the dynamics of organic matter and nutrient cycling^4^. Soil microbial biomass is known to reduce with depth due, in part, to the limited availability of organic matter^4,5^ and change in the physical and chemical properties at different soil horizons. Van Leeuwen et al. (2017)^4^ stated however, that horizons below 30 cm depth might contain up to 30% of fungal and bacterial biomass, and soil microbial communities in the upper horizons are affected mostly by land use while pedological parameters affect it the most in deep horizons. Similarly, Mann, Bass, and Meadows (1990)^6^ considered the depth of burial to have the most effect on decomposition, together with temperature and access by insects. The impact of sampling depth on soil bacterial and fungal community composition relative to decomposing pig remains was established, albeit in an experimental microcosm^7–9^. Thus, it is important to study microbial community changes in upper versus deeper soil horizons. The question of how soil microbial community structure in the different soil layers may be affected by carrion decomposition, and how these differences might be exploited for forensic applications such as the estimation of time since death, must be addressed. Furthermore, the depth of burial relative to sample collection in crime scenes is important, particularly for the location of clandestine graves and potential translocation of remains.

The current experiment was conducted to further understand the impacts of the decomposing cadaver on soil microbial communities at different depths. Specifically, microcosms were used to test the soil depth that is affected by decomposition of the common house mouse (*Mus musculus*) as a human surrogate^10,11^. In addition, the effect of sampling on soil microbial community and elemental composition was assessed. Murine models were monitored during aboveground and subsurface decomposition to test the reliability of potential clandestine grave location using a combination of molecular microbial ecology and relevant physicochemical analyses. This experiment was designed to test the hypothesis that soil microbial community associated with *M. musculus* decomposition will differ depending on whether carrion deposition was aboveground or in the subsurface.

## Results

### Soil bacterial necrobiome metabarcoding

Skeletonization of the animal models was observed within the first three months of the study. As a result, only soil samples from the first 90 days (months 0–3) were sequenced.

**Fig. 1:**
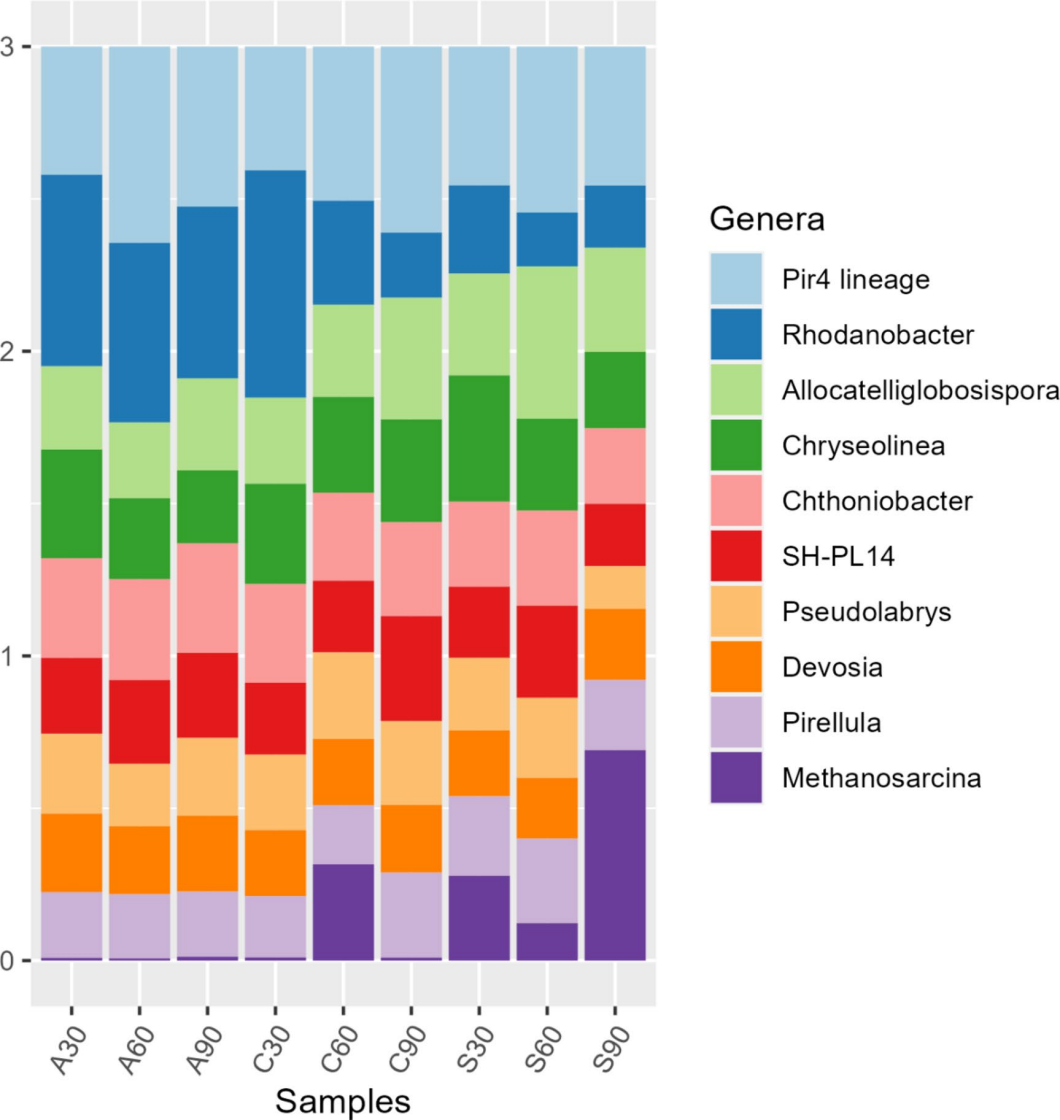
Genus-level bacterial resolution bar-plot of the control (C), subsurface (S) and aboveground (A) decomposing-mice associated soils at 30 cm deep on days 30, 60 and 90.

The top ten predominant bacterial phyla across the three treatments and study duration were Proteobacteria, Planctomycetota, Bacteroidota, Actinobacteriota, Acidobacteriota, Chloroflexi, Verrucomicrobiota, Myxococcota, Halobacterota and Bdellovibrionota, while Euryarchaeota was predominant in aboveground mice soils only (Supplementary Figure S1). Resolution at genus level showed the top 10 distinguishable taxa as Pir4 lineae, Rhodanobacter, Allocateliglobosispora, Chryseolinea, Chthoniobacter, SH-PL 14, Pseudolabrys, Devosia, Pirellula, and Methanosarcina. Methanosarcina showed the highest increase in abundance for the subsurface soils at the postburial interval of 90 days (Fig. 1).

**Fig. 2:**
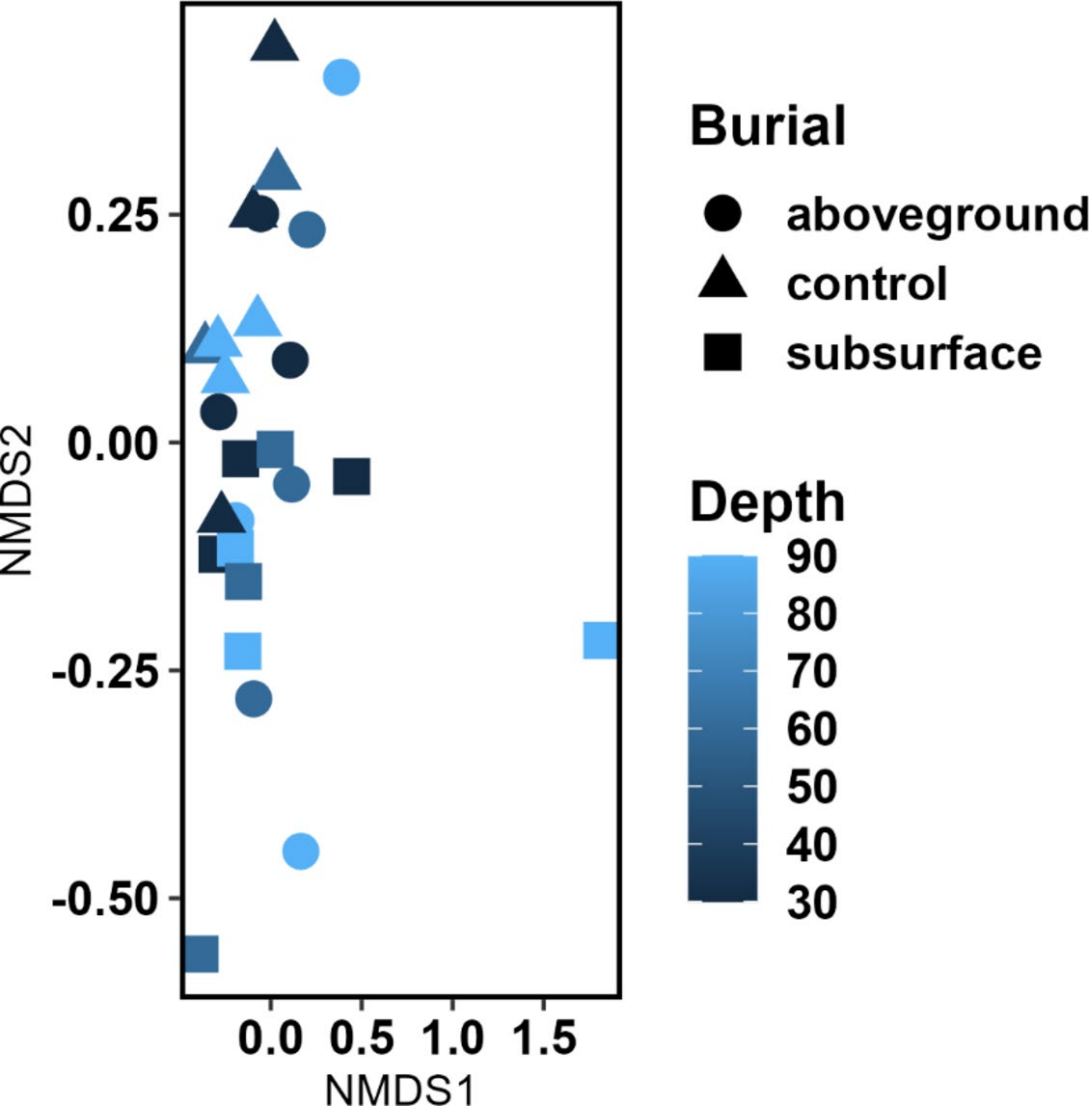
NMDS (Stress = 0.1068477) bacterial community analysis of the control (black filled triangle), subsurface (black filled square) and aboveground (black filled circle) decomposing-mice associated soils at 30 cm deep on days 30, 60 and 90.

**Fig. 3:**
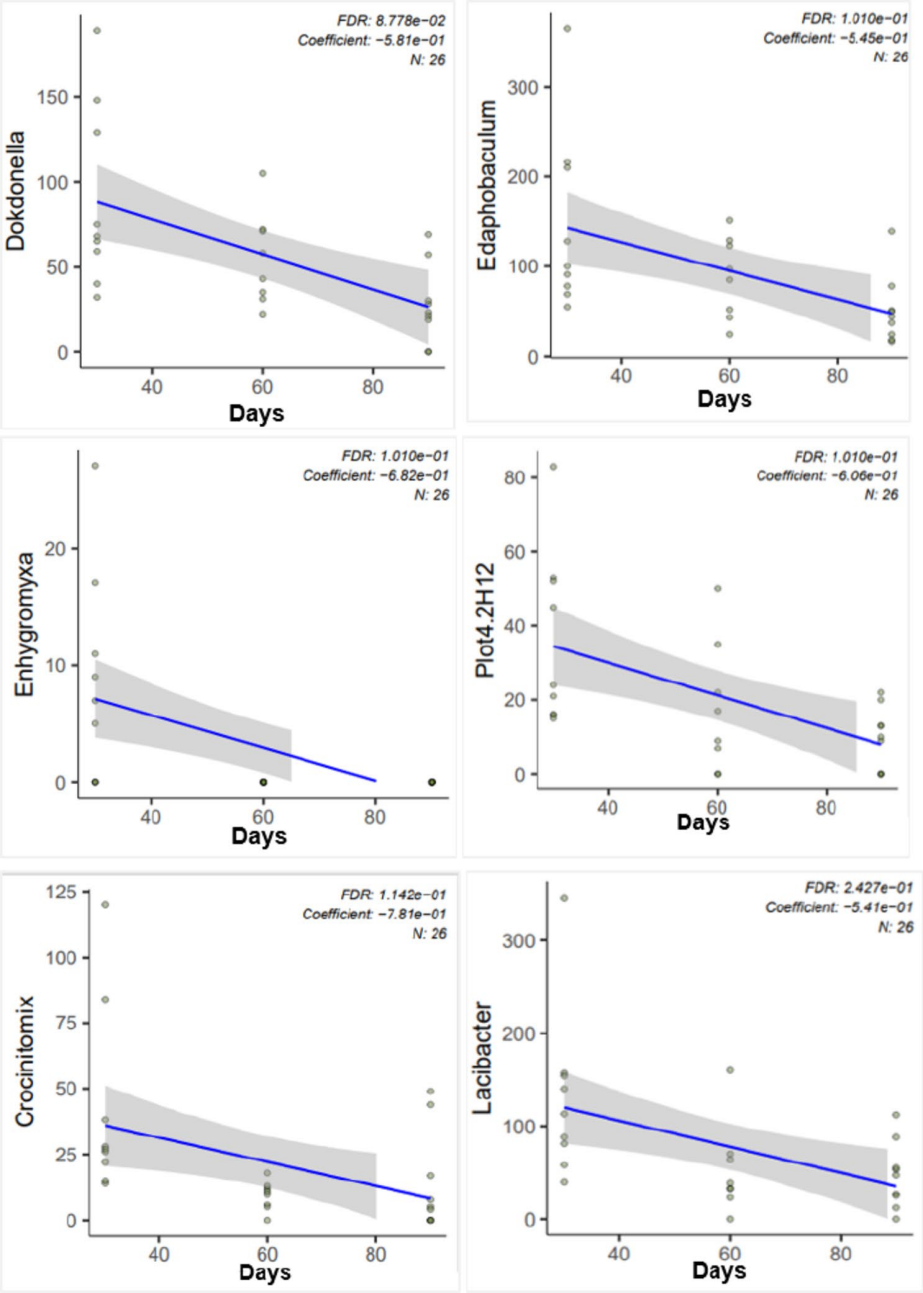
Average (*n* = 9) MasLin2 analysis showing numerically abundant genera from the soil bacterial metagenome on days 30, 60 and 90 of decomposition.

**Fig. 4:**
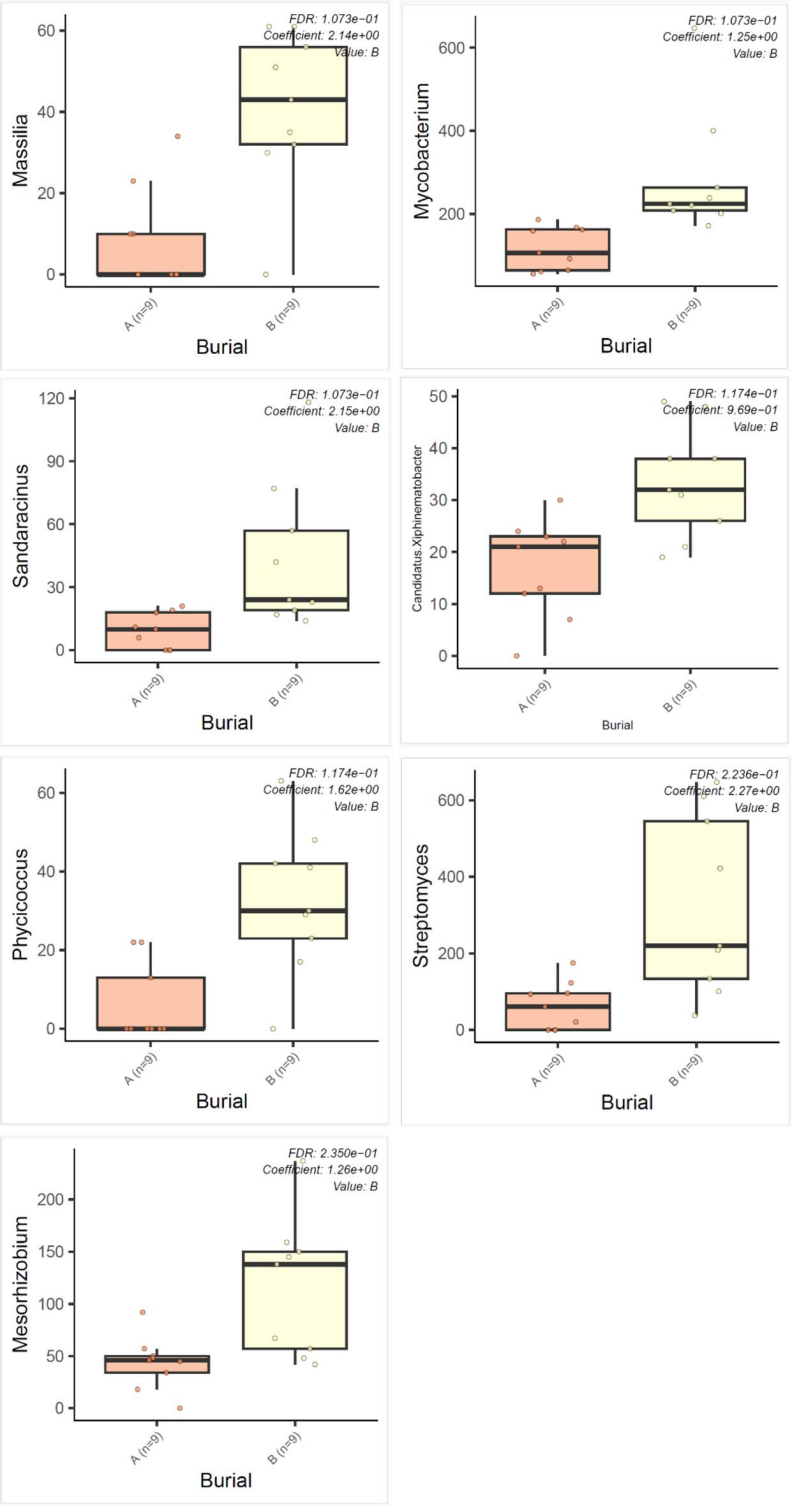
Average (*n* = 9) MasLin2 analysis showing differences in numerically abundant genera from the soil bacterial metagenome in response to *M. musculus* deposition aboveground (**A**) vs. subsurface (**B**).

An NMDS stress = 0.1068477 was recorded for phyla abundance for the control, aboveground and subsurface deposition microcosms, and sampling time (Fig. 2). Dissimilarity test ANOSIM indicated that mouse deposition aboveground and in the subsurface was significantly different (*p* = 0.0082). In contrast, the decomposition or sampling time was not statistically significant across the study treatments (*p* = 0.5382). MaAsLin2 analysis identified specific genera that were statistically significant relative to time and they included Dokdonella, Edaphobaculum, Enhygromyxa, Plot4.2H12, Crocinitomix and Lacibacter (Fig. 3). The abundances of Massilia, Mycobacterium, Sandaracinus, Candidatus xiphinematobacter, Phycicoccus, Streptomyces and Mesorhizobium genera increased significantly aboveground (Fig. 4). Lastly, Variovorax occurrence was significantly higher in the control soils (*p* = 0.000027) than the treatments with mice deposition aboveground and in the subsurface. The Shannon index returned no significant difference for soil bacterial community alpha diversity between the control, aboveground and subsurface mice deposition (*p* = 0.0933) and sampling times (*p* = 0.1333) (Supplementary Figure S2, A-D). Community richness was significantly different for aboveground vs. subsurface decomposition and control soil (*p* = 0.0011) but not in response to sampling time (*p* = 0.2135).

Diversity profiling with denaturing gradient gel electrophoresis (DGGE) showed that shifts in bacterial 16S rRNA and fungal 18S rRNA genes distinguished between control and mice microcosms over the 1-year period (May 2017 – May 2018). However, this low-resolution technique implied that the bacterial communities did not differentiate between aboveground vs. subsurface deposition of carrion (Data not shown). As also demonstrated by Olakanye et al. (2017)^12^ and Bisker et al. (2021)^13^ with metagenomics metabarcoding in an piglet-based study, future work should explore whether the soil microbial community can provide clues for aboveground vs. subsurface clandestine grave location in the long term.

### Soil pH trends

The average (*n* = 3) pH values for the aboveground, subsurface and control soils were measured and compared over 360 days (12 months). With the exception of the aboveground *M. musculus* soils on day 210, the overall pH trends were comparable for the control and experimental treatments throughout the study (Supplementary Figure S3). Two-way ANOVA did not show significant differences between the A, S and C microcosms (*p* = 0.17) throughout the study.

### Temperature, accumulated degree days (ADD) and weather conditions

Weather data for Middlesbrough U.K. were downloaded from www.worldweatheronline.com while soil temperatures and humidity were recorded continuously throughout the 1-year study using probe data-loggers, and then further expressed in accumulated degree-days (ADD) (Supplementary Figure S4). Atmospheric temperatures recorded for spring to summer 2017 encompassing days 30, 60 and 90 as the focus for this report, increased from 13 °C on day 1 to 16 °C on day 90. Soil temperatures during this period started from 11.63 ± 0.11 °C (control), 11.75 ± 0.12 °C (aboveground) and 11.7 ± 0.11 °C (subsurface) on day 1 (ADD10.97) and increased, becoming more comparable atmospheric conditions, to 16.96 ± 0.12 °C (control), 17.62 ± 0.06 °C (aboveground) and 17.7 ± 0.15 °C (subsurface), on day 90 (ADD1425.72). Two-way ANOVA showed significant differences in soil temperature (*p <* 0.05) between the control and experimental treatments.

### Total body scoring (TBS)

**Fig. 5:**
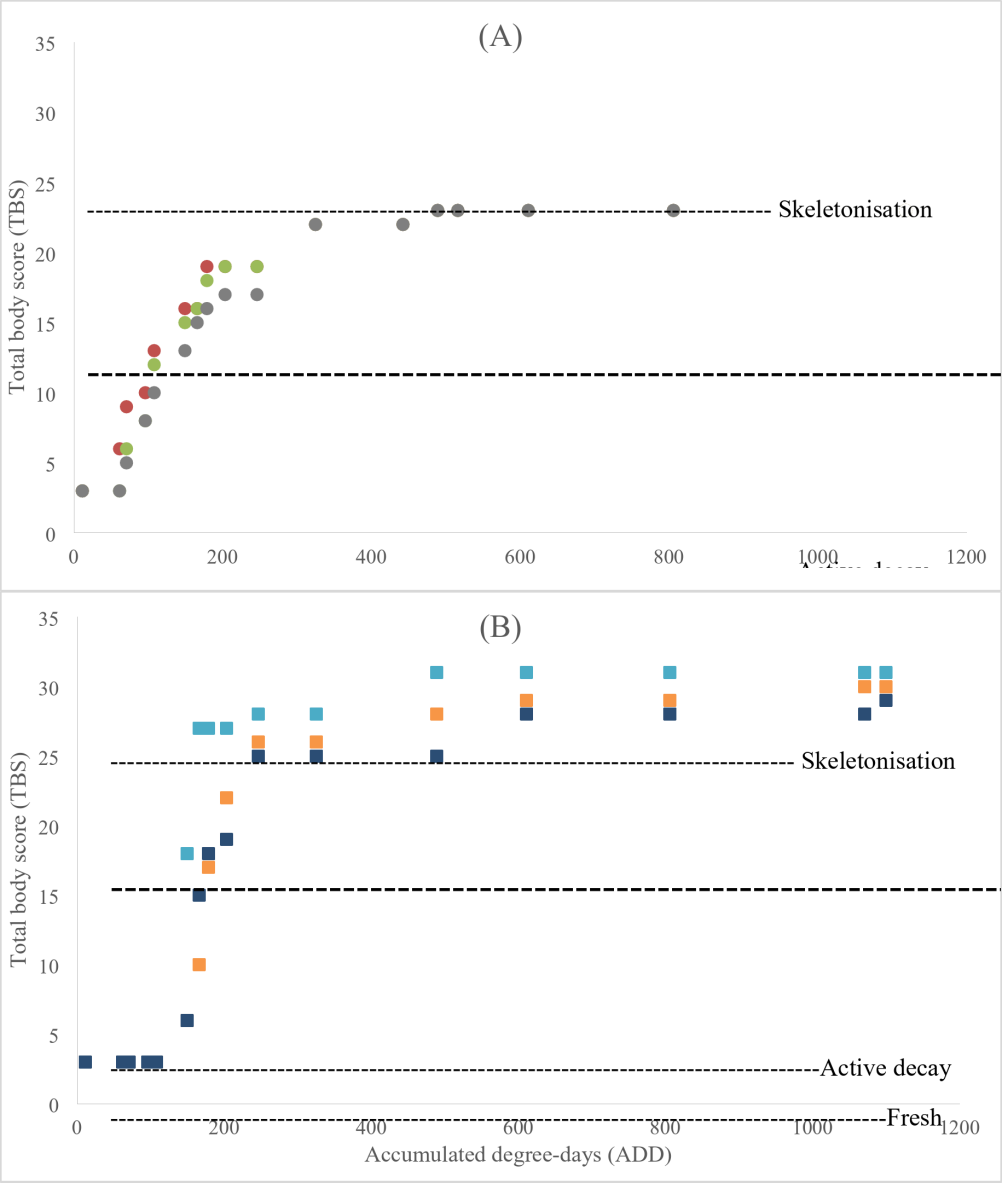
Total body scores of mouse 1 (brown filled circle), mouse 2 (green filled circle) and mouse 3 (gray filled circle) decomposing subsurface in transparent pipes (**A**); mouse 4 (blue filled square), mouse 5 (orange filled square) and mouse 6 (violet filled square) decomposing aboveground (**B**).

The transparent pipes used for subsurface TBS observations do not reflect what happens in brown pipes because of differences in temperature and the effect of sunlight, which were probably higher in the transparent pipes. As a result, accumulated degree days were used to infer decomposition in brown pipes. Total body scores for the aboveground and subsurface decomposing mice were generated (Fig. 5) based on the approach described by Megyesi, Nawrocki, and Haskell (2005)^14^ and modified by Adlam and Simmons (2007)^15^.

Although a faster decomposition rate was recorded for aboveground mice than those in the subsurface, mice decomposing aboveground remained in the fresh stage longer (TBS = 3; days 1–10) than those in the subsurface (Table 1). Active decay occurred between days 13 and 20 while advanced decay was between days 20 and 52. Insects such as calliphorid and sarcophagid flies were observed on the aboveground mice on day 1/ADD 11 and day 13/ADD 149 leading to an infestation with blowfly maggots. On day 34/ADD 489, newly hatched flies were observed on soil and around the aboveground mice carcasses. Not all aboveground triplicates decomposed in a similar pattern hence mouse 6 appeared fresh for longer than mice 4 and 5, which were consumed rapidly by maggots. Subsequently, one mouse was scavenged completely leaving only its fur and exposed bones. Skeletonization started from days 20 (TBS 25) to 52 for the aboveground mice while it was not possible to observe this stage of decomposition on subsurface mice.

Mice decomposition in the subsurface went through the fresh stage quickly up to advanced decay stage, and followed by a decrease in decomposition rate. Specifically, active decay happened between days 7 and 13. On day 34/ADD 489 moss started forming on the inner part of the transparent pipes and became dense on day 52/ADD 806 after which no observation was possible. Nevertheless, the three mice had a TBS score of 23/ADD 324 by then. Overall, the TBS results suggest that release of nutrients into the soils happened during active decay and skin rapture (days 7–13; subsurface; days 13–20, aboveground).

**Table 1:** Total body score (TBS) range from 0–30 and indicate the level of carcass decay across four decomposition stages: fresh (0–3), active (3–5), advanced (5–17) and skeletonized (> 25).

Time (days)	ADD	Average aboveground TBS	Average subsurface TBS
1	11.28	3	3
10	107.84	3	11
15	178.75	20	17
24	324.71	26	22
34	489.01	28	23
42	610.88	29	23
52	805.97	29	23
71	1100.1	30	/

### Soil mineral composition

**Fig. 6:**
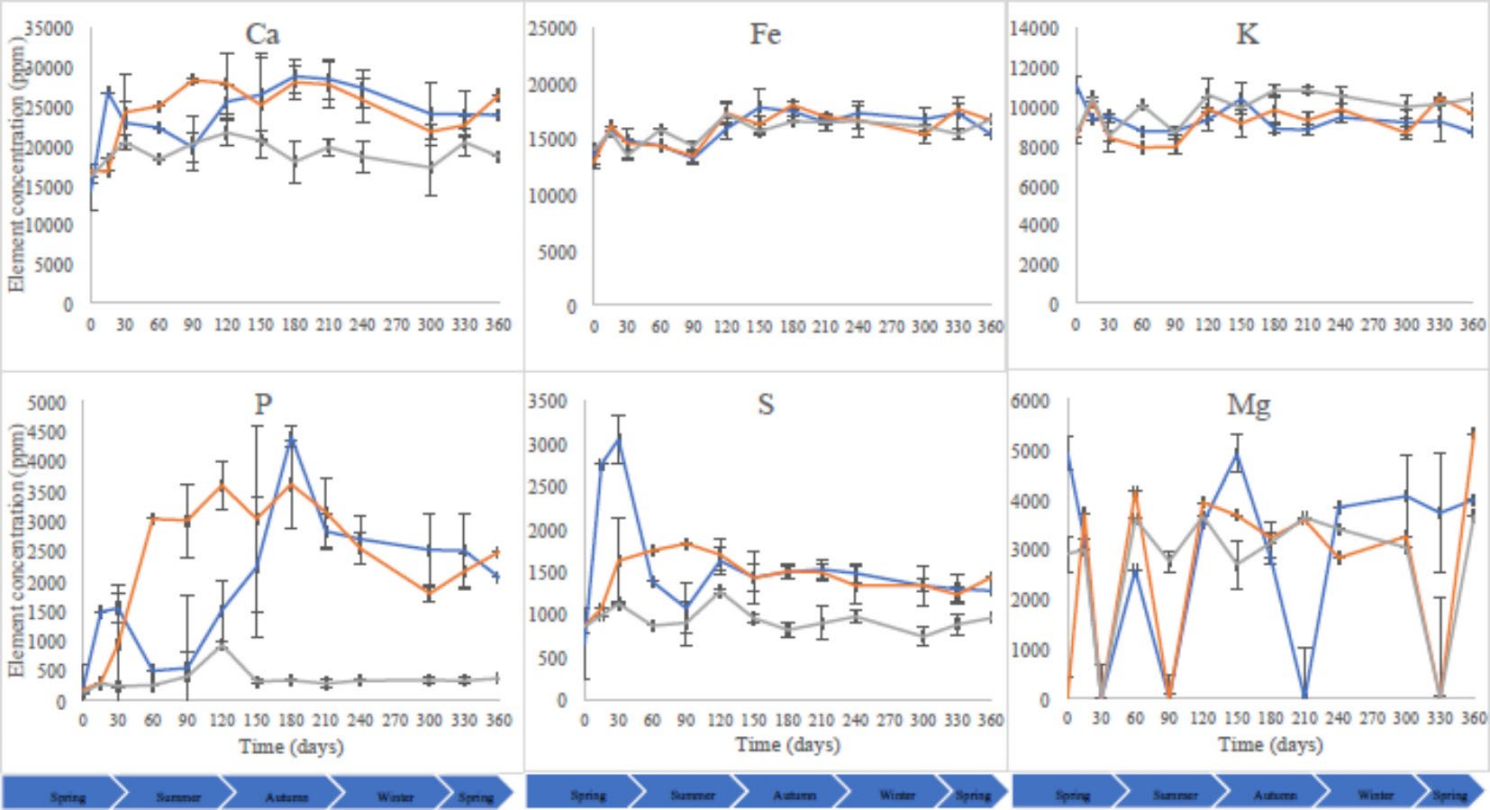
Average (n = 3) soil composition in iron (Fe), potassium (K), calcium (Ca), phosphorus (P), sulphur (S) and magnesium (Mg) as measured by pXRF for control (blue filled square), aboveground (gray filled square) and subsurface (orange filled square) decomposing M. musculus treatments. Each datum point is the mean of triplicate samples. Error bars are SEM.

Soil mineral Ca, Fe, K, P, Mg and S were monitored for their role in biological functions, nutrient recycling and microbial activity and importance as biomarkers of decomposition products. Figure 6 shows the patterns in the composition of these elements during for the control, aboveground and subsurface mice soils. Calcium concentration for the aboveground mice treatment soil was marked by a minor increase throughout the study starting at 16 098 ± 819 on day 1 (spring 2017) to 18 633 ± 1670 on day 360 (month 12; spring 2018). The control and subsurface treatments calcium concentrations increased from day 1 (14 321, control; 16 776, subsurface mice) until day 360 (23 882, control; 26 375, subsurface mice). Two-way ANOVA detected significant differences in calcium concentration between the control and experimental aboveground and subsurface mice soils throughout the study. Overall, the increase in soil calcium concentration was more pronounced in response to subsurface than aboveground decomposition.

There was an increase in soil potassium content on day 15 (10 245 ± 345, subsurface; 10 463 ± 153, aboveground mice) followed by a decrease on day 30/month 1 (8 400 ± 733, subsurface; 8 491 ± 301, aboveground mice). Further analysis indicated a negative correlation in potassium (K) content between control and aboveground mice soils and no correlation between the control and subsurface decomposition treatments. This was in contrast to a positive correlation observed between the experimental treatments, which had comparable patterns throughout the study that were distinct from control soils. Two-way ANOVA indicated a statistically significant difference between the different treatments.

Also, there were significant differences in soil Fe, P, K and S composition between the different treatment groups (*p* < 0.05) but no significant difference in soil elemental composition between the different time points. In addition, although two-way ANOVA revealed no significant temporal differences in Mg composition between the different treatments (*p* > 0.05), significant changes were recorded at different time-points for this mineral.

### Effect of sampling on soil elemental profiles

The experimental treatments where *M. musculus* decomposition was monitored aboveground and in the subsurface, were replicated (A2; S2) to assess the effects of sampling on soil elemental composition^13^. The results obtained for sampling only on days 0 and 360 for the different replicates had patterns similar to those described for the treatments sampled at regular intervals (Supplementary Figure S5). Two-way ANOVA did not detect any significant differences in soil elemental composition between the replicates.

## Discussion

### Bacterial community resolution

Although different mammalian models were used, the dominance of phyla such as Proteobacteria, Planctomycetota, Bacteroidota, Actinobacteriota, Acidobacteriota, Chloroflexi and Verrucomicrobiota are in accordance with those reported in the literature for aboveground^16,17^ and subsurface decomposition^18^. For example, Chloroflexi, a clade of multicellular, filamentous and anoxygenic photosynthetic bacteria^19^ that use hydrogen sulphide as a reductant, were more abundant in the presence of mice aboveground and in the subsurface than control soils. Although generally characteristic of anaerobic conditions, hydrogen sulphide is also indicative of the putrefactive phase of decomposition accounting for its increase in the presence of *M. Musculus*. Trends for the relative abundance of Chloroflexi in soil in the absence of cadaver was similar to findings reported by Singh et al. (2018)^20^ where the occurrence of this taxon increased at 1 and 5 m distances away from human cadavers that were decomposing aboveground.

A higher taxonomic resolution allowed the identification of the ten most predominant genera. Overall, the NMDS stress of 0.1068477 showed a good overview that the recorded differences in taxa abundance were a result of the absence (control soil only) vs. presence of *M. musculus*, aboveground vs. subsurface deposition, and sampling time. Statistically significant differences in abundance at 30 cm depth, as determined by MaAsLin2, identified target microbial clock indicators at genus level which provided differentiation for: decomposition time (Dokdonella, Edaphobaculum, Enhygromyxa, Plot4.2H12, Crocinitomix and Lacibacter); deposition locales of aboveground vs. subsurface (Massilia, Mycobacterium, Sandaracinus, Candidatus xiphinematobacter, Phycicoccus, Streptomyces and Mesorhizobium); and control soil with no remains (Variovorax). Most of the statistically significant temporal indicators are Gram-negative aerobic rods. Dokdonella, strict aerobes first isolated from soil (Yoon et al., 2006)^21^, and Edaphobaculum are non-motile while Enhygromyxa and Crocinitomix are motile gliders. Also, Enhygromyxa are typically halotolerant and halophilic members of the Nannocystaceae family (Garcia and Müller, 2014)^22^. Lacibacter and Crocinitomix belong to the Bacteroidota phylum, where Crocinitomix are characterised as yellow-pigmented and cold-adapted strict aerobes (Bowman et al., 2003)^23^. Their distribution profile in Fig. 3 infers that their occurrence was potentially linked to the mice carrion that were deposited into the microcosms whilst frozen.

As differentiators between decomposition aboveground and in the subsurface, Gram-positive rods Mycobacterium are Actinobacteiota that typically include tuberculosis- and leprosy-causing pathogens. Non-tuberculous mycobacteria consist of slow and fast growing populations (Tortoli, 2019)^24^. Sandaracinus degrade starch (Mohr et al., 2012)^25^ while nematode-associated Candidatus xiphinematobacter inferred arthropod-microbiome interactions within our study treatments. The mesophilic Gram-positive and aerobic Phycicoccus (Lee, 2006)^26^, filamentous Gram-positive aerobic and multicellular Streptomyces which are characteristic of soils and decaying plant litter (Otani et al., 2022)^27^ and Gram-negative nitrogen-fixing Mesorhizobium indicated further the environmental parameter-microbiome interactions particularly in the presence of decomposing *M. musculus*. The aboveground vs. subsurface indicators will be useful especially when remains have been translocated from the primary locales within the first 90 days of deposition. Members of the Variovorax genus were indicators for pristine soil with no remains, despite the proximity of the microcosms for this study. Therefore, they are potential markers for the detection and discrimination of non-gravesoils compared to both subsurface and aboveground decomposition. Lastly, the anaerobic methanogenic *Methanosarcina* genus, with some members being halophilic, largely characterised the subsurface mice treatments compared to control and aboveground soils at 90 days. As a result, this genus can be used as a marker of decomposition mainly in the subsurface for the detection of gravesoil. In addition, its anaerobiosis would distinguish the provenance of soil (surface vs. subsurface) and give indications on whether a location/soil had been dug out.

Our results were obtained using mice decomposing above or beneath topsoil. Deterministic taxa markers for aboveground and subsurface decomposition must be explored further. A similar comparative study conducted in situ is essential to confirm the current findings and validate the approach for use in real forensic cases. Larger human proxies such as pigs would ensure further proof-of-concept investigations in different soils, weather and environmental conditions with more replicates for increased statistical power. Ultimately, the microbial markers and clock indicators of gravesoil identified in the current work, particularly at the genus level, require further exploration based on donated human cadavers. This would confirm the applicability of our findings to human cadavers and the usefulness of the markers identified for aboveground and subsurface gravesoil discrimination. Future work can also consider total profiling of the necrobiome for mammalian proxy parallel to that of the proxy + gravesoil.

The main advantage of using animal models in decomposition research is the possibility to replicate studies and control the cadaver’s ante-, peri- and post-mortem circumstances together with environmental conditions, which leads to an increase in statistical power compared to relying on donated human bodies. *Mus musculus* was chosen as a human analogue for the current study mainly for its small size and fast decomposition, allowing for statistically accepted triplicates and methodological robustness. Also, Takeuchi et al. (2011)^10^ reported the similarity between human and mice skin permeability, and analogous biochemical, cellular, and physiological pathways while Nguyen et al. (2015)^11^ highlighted their suitability for biomedical research due to their similarity with humans physiologically and anatomically, especially in the composition of their gastrointestinal tract. Nevertheless, the dissimilarities in diet, living environment, genetic background, physiology and microbiome, may influence murine and human decomposition processes^11^, hence direct comparisons between animal models and human beings remain a concern. Only female mice were selected for the study to account for inter-sex variability. According to Javan et al. (2016)^28^, the succession pattern in humans is highly comparable within the same sex and differs significantly between the two sexes where the researchers recorded Pseudomonas predominance in female cadavers and Streptococcus in males.

The experimental design was cognizant of the implications of using frozen carrion^29,30^. Hence thawing was allowed to happen directly within the microcosms to achieve consistent temporal impacts of the decay process on the study gravesoils. Although topsoil and PVC tubes afforded a controlled proof-of-concept study, in situ burials should be adopted in future research. These will test and reflect better the local environmental and biochemical conditions relating to depth-dependent changes in mineralogy, soil structure, organic matter, nutrient concentrations and indigenous microbial communities at crime scenes. Such investigations will determine the transferability of the current findings to real forensic contexts.

Detecting patterns of change in soil bacterial communities, which represent up to 95% of soil biomass^4^, has recognised potential to indicate time since death and detect clandestine graves. These successive patterns can also confirm that decomposition happened in a soil without the physical presence of a cadaver, as in translocation cases where remains are moved to a secondary burial site to hide evidence. During this *M. musculus* study, MaAsLin2 analysis of the metagenome showed increased abundances of specific genera in the control soil, relative to sampling or decomposition time, and with statistical differences between aboveground and subsurface depositions. The hypothesis that the soil microbial community will differ between *M. musculus* decomposition aboveground and the subsurface is accepted for the 30 cm depth and up to 90 days of decay.

### Environmental conditions

Decomposition rates and processes are influenced by weather conditions^31–35^, especially temperature, which was reported to have a major impact on biological function and microbial activity^32,36^. Therefore, atmospheric temperature records were downloaded from www.worldweatheronline.com and compared to data recorded on site using data loggers. The similarities, particularly on day 90 as the endpoint for this report, in atmospheric (16 °C) and soil temperatures (C = 16.96 ± 0.12 °C; A = 17.62 ± 0.06 °C; S = 17.7 ± 0.15 °C) suggests the feasibility of using temperature records from meteorological stations to estimate time since death and decomposition stage. While measuring temperature in situ gives accurate results for fundamental research, meteorological station records would be highly beneficial in real case scenarios where forensic scientists do not have in situ temperature records.

Soil pH from cadaver decomposition islands is typically responsive to different stages of decay, with soil layers immediately next to carrion remains fluctuating due to the concentrations of ammoniacal compounds, for example. Recorded shifts in pH within the 30 cm depth were not statistically significant between the control and experimental treatments potentially due to the decomposing *M. musculus* biomass relative to the soil volume. This was in contrast to previous work where destructively sampled microcosms consisting of 1:20 (w/w) ratios of *Sus scrofa* or plant litter biomass (4 g) to fresh weight soil (80 g) showed statistically significant pH differences between control and experimental treatments^37^.

### Total body scoring

Most of the decomposition happened during the first two months due to the increase in temperature during summer, and was in accordance with findings in the literature^38,39^. Generally, aboveground mice went through the different decomposition stages faster than those in the subsurface. Also, blow flies and maggots were observed on two of the aboveground mice. Subsequently, members of this group that were not eaten by blowfly maggots were desiccated which potentially slowed down their decomposition. This is in accordance with Galloway et al. (1989)^40^ who reported a delayed oviposition in dry cadavers. Our findings align with previous discourse that decomposition in subsurface settings is usually slower compared to aboveground settings due to lower temperatures and limited insect and scavenger access to cadavers^6,41^. The observed blow flies, maggots, dessication and plant growth will have reduced further the decomposing biomass with subsequent impacts on exogenous properties such as soil pH, as well as bacterial community profiles between aboveground and subsurface deposition. While experimental triplicates are recognised as statistically and mathematically robust, more replicates should be considered as per Metcalf et al. (2013)^42^ and Liu et al. (2023)^43^ for higher statistical power and to account for situations where the decomposition trend for one of triplicate animal models deviates from the other two.

Transparent and brown U-PVC pipes were used to visually assess aboveground vs. subsurface total body scores. Therefore, ADD calculations were used in combination with TBS to account for differences in decomposition rates that resulted from unique environmental conditions of soil temperature and exposure to direct sunlight within the two types of pipes. Specifically, positioning the subsurface mice on the front side of the transparent pipes for visibility would increase the rate of decomposition compared to the subsurface group within brown pipes. Cadavers persisted for different intervals after the start of putrefaction and had significantly different decomposition patterns and rates. Consequently, the calculated total body scores showed high variability in decomposition rates between replicates decomposing aboveground even at the same ADD, under the same environmental conditions, which highlights the challenge of estimating time since death outdoors during aboveground decomposition. Nevertheless, Table 1 shows the applicability of ADD instead of time in days or months in comparing two different decomposition settings of aboveground vs. subsurface. As shown in the current findings, decomposition stages vary at similar PMI/PBI thus a juxtaposition of ADD and TBS allowed for further reliability within the comparisons.

### Soil mineral composition

Our results suggest that the release of decomposition fluids and, therefore, influx of nutrients into soil happened before day 15 causing the considerable increase in soil potassium concentration. However, these nutrients are generally used by soil microorganisms and plants^44–46^ which probably explains the subsequent decrease on day 30/month 1. These trends highlight the potential of using the influx of potassium into the soil^47^ during the early putrefactive stages as an indicator of the phase of decomposition or presence of cadaver even if the body has been removed. If confirmed by further case studies, this could help detect decomposition soils and confirm suspected recent clandestine graves or decomposition sites.

Most of the research conducted on soil as trace evidence was hitherto based on either physicochemical or microbiological profiling^48–51^. The accuracy and adoption of microbial-based forensic science must be underpinned by the application of currently used bio- and physico-chemical methods giving more validation for scrutiny in court proceeding. Due to its simplicity and portability, portable X-Ray fluorescence (pXRF) has become one of the most common techniques for elemental analysis. This rapid, non-destructive, simultaneous multi-element technique^52^ is readily available in forensic laboratories and used for routine analysis of trace evidence and artefacts, which makes the expansion of its use to complement microbial community profiling of gravesoil, within forensic microbial ecology, viable and cost-effective for forensic units. pXRF is affected by factors such as soil moisture content and heterogeneity hence the importance of sample preparation prior to analysis^53^.

Soil samples were analysed by pXRF to assess the changes in soil elemental composition. The aim was to illustrate the importance of physicochemical characterisation of gravesoil, with pXRF as an example technique, to complement microbial ecology techniques in a forensic context. Phosphorus (K), magnesium (Mg), calcium (Ca), potassium (P), iron (Fe) and sulphur (S) were selected for their importance in biological functions, nutrient recycling and microbial activity. Sulphur is found in protein-forming amino acids such as methionine and cysteine, which are degraded by microbial-driven decomposition to form volatile sulphur compounds that attract necrofauna insects^54–56^. Potassium is important for muscles and the nervous system functioning and has been used for the estimation of time since death based on its concentration in the vitreous humour^57,58^ and synovial fluid^59^. In addition, it is present in soil and plays an important role in plant growth and metabolism^60^. Likewise, phosphorous, magnesium and calcium are important elements for biological functions and soil nutrients. Phosphorous concentration was reported to change during decomposition^61–64^ while calcium changes underneath bison carcasses were detectable up to 7 years after interment^65^. Furthermore, calcium, phosphorous, potassium and magnesium are major minerals in the human body with blood concentrations dropping from 8.45 to 3.70 mg dL^− 1^ for calcium and increasing from 100.72 to 188.49 µg dL^− 1^ for iron after 264 h of putrefaction^66^. Sulphur and iron are trace elements necessary for biochemical functions of the human body.

Overall, the current study findings suggest that the effect of nutrients such as potassium is time limited as it is released mainly after the rupture of the cadaver skin through the liquefaction of tissues^47^. Therefore, pXRF is a key example of the established physicochemical approaches that can be used in holistic forensic investigations where soil potassium concentration could, for example, help assess the time of skin rupture and release of nutrients into the surrounding cadaver decomposition island.

### Aboveground vs. subsurface decomposition

A pronounced increase in soil calcium was recorded when mice were deposited and decomposed in the subsurface than aboveground. One likely explanation is the exposure of mice during aboveground decomposition where their desiccation or heavy consumption by maggots^65^ reduced the volume and extent of the cadaver decomposition island^67,68^ and, subsequently, the amount of calcium released into the surrounding soil. However, and despite the statistically significant differences between the different treatments, control soils had an increase in soil calcium comparable in magnitude to the experimental soils. This might have been due to sampling depth at 30 cm below the small-sized decomposing mice where the change in calcium concentrations might not have reached. Also, mice contribution to soil calcium was potentially minor due to their small size hence the undetected changes to this particular mineral. Some soil elements such as magnesium were indicative of different sampling times but did not differentiate between aboveground and subsurface decomposition.

### Effect of regular sampling on soil elemental profiles

The effect of regular sampling^13^ on soil elemental composition during the decomposition of *M. musculus* was assessed by replicating the experimental aboveground and subsurface treatments as A2 and S2. The first set of triplicates (A and S) was sampled monthly, while the second set (A2 and S2) was sampled only on the first and last day of the study. Two-way ANOVA did not detect any statistically significant differences in elemental composition profiles between the two sets of treatments. The similarity in patterns suggests that the effect of sampling on soil elemental composition is negligible in this specific case, for a study that was set outdoors under ambient weather conditions. Studies under controlled conditions would be essential to assess the impact of sampling in detail. Similarly, additional aboveground vs. subsurface case studies set under different outdoor conditions using larger animal models and human cadavers are required to test, confirm and validate the current findings to much wider contexts.

## Conclusions

This study is, to our knowledge, the first to combine elemental and molecular microbial analyses specifically to inform aboveground and subsurface decomposition in a forensic context. Since there was no significant difference in elemental profiles between the aboveground vs. subsurface *M. musculus* burials, but between control and experimental soils particularly for calcium, we propose that pXRF can complement soil necrobiome profiling to identify gravesoils in general. Spikes in calcium could also detect gravesoil/crime scenes in cases of cadaver translocation. Adopting this approach to real-life investigations necessitates further research and case studies preferably using human remains, especially since differences exist in decomposition rates and processes between human and animal cadavers.

## Methods

To mitigate for space limitations and inimical impacts on the university campus soil ecosystem, and to achieve homogeneity for a proof-of-concept study, commercial multi-purpose topsoil bags were sourced from Wickes^®^ (Northampton, UK), and used for the mice decomposition microcosms. The topsoil was left outdoors at Teesside University (Lat. 54.572426°N; Long.1.236004°W) to equilibrate for two weeks and mixed thoroughly to ensure homogeneity prior to setting up the experiment.

### Experimental design

**Fig. 7:**
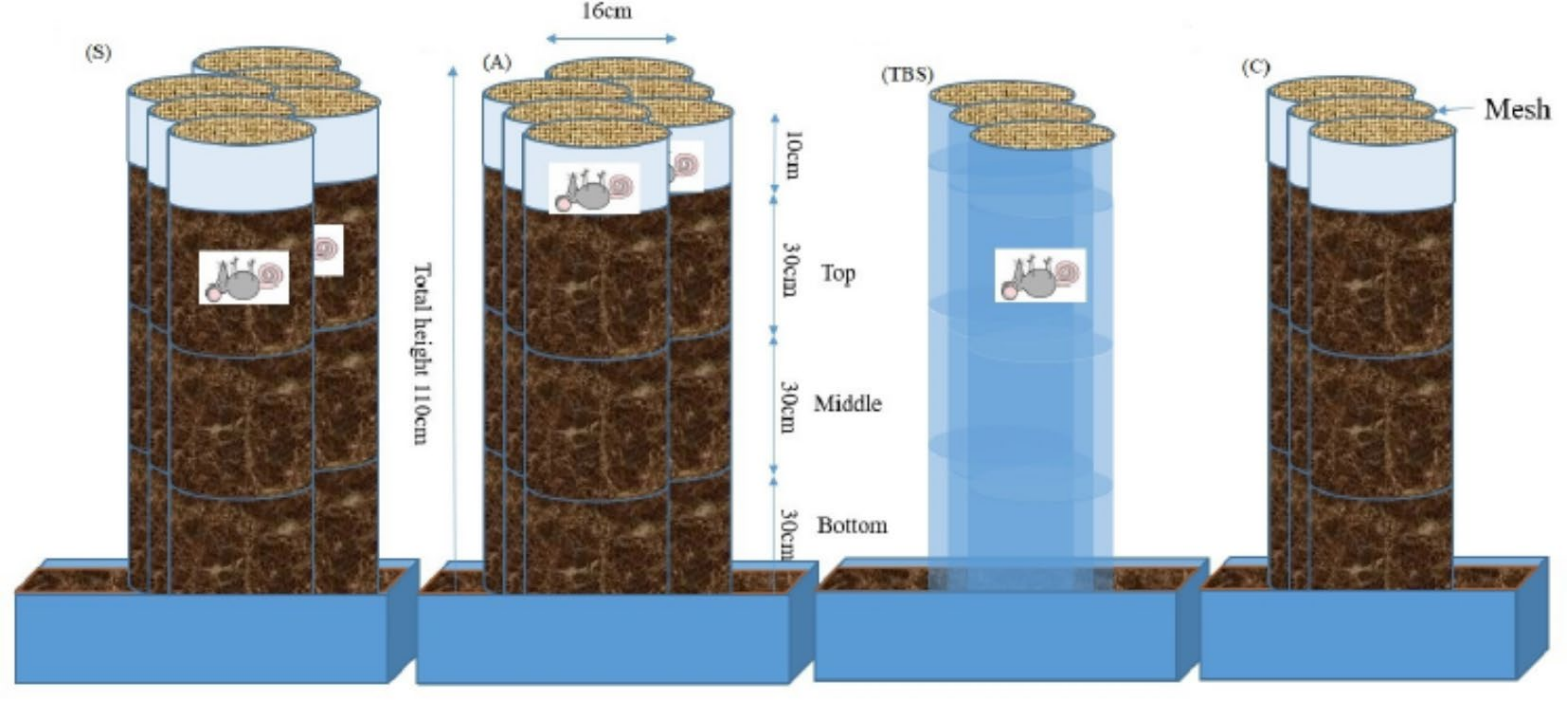
Experimental design of the decomposition study with triplicate microcosms of 13 kg soil and mice decomposing aboveground (A), at 10 cm in the subsurface (S), in transparent pipes for total body score (TBS), and soil-only controls (C).

The 360-day study was conducted outdoors from Spring (May 2017) at Teesside University crime scene house, Middlesbrough, U.K. To achieve requisite differences in soil depths, brown U-PVC pipes filled with ≈ 13 kg of the equilibrated commercial topsoil each were placed in half-filled perforated topsoil boxes (L60xW40xH27 cm) to limit eluviation. Female mice (*Mus musculus*; ≈13 g) for the study were obtained frozen from John’s Place4Pets (Middlesbrough, U.K.); therefore, the experiment did not use live vertebrates. Notwithstanding this, the appropriate ethical approval was awarded by the School of Science and Engineering, of Teesside University, U.K. No thawing was performed prior to burial to minimise contamination with exogenous microbes, and to ensure that any thawing-induced shifts in the mice necrobiome^26^ occurred within the burial scenario, impacting the gravesoil as consistently as feasible. Individual mice were placed to decompose either aboveground (A) or in the subsurface (S) (Fig. 7; Supplementary Figure S6).

The first set of microcosms consisted of three brown U-PVC pipes with one *M. musculus* each decomposing aboveground (0 cm; A). For the second set, triplicate treatment brown U-PVC pipes were used to bury individual *M. musculus* each at 10 cm subsurface (S). A final set of three brown U-PVC pipes was used for soil-only controls (C). Identical triplicate aboveground (A2) and subsurface (S2) experiments were set up in brown U-PVC pipes and run simultaneously, and sampled only on day 0 and day 360 of the experiment to assess the effect of regular sampling on soil elemental and microbial composition. Soil samples (≈ 25 g) were collected from the microcosms on days 1 and 15, and then regular 30-day intervals up to 360 days at carcass-level (0–10 cm), 30, 60, and 90 cm depths using stainless-steel spatulas, and stored in sterile universal tubes (Sarstedt, Australia). Spatulas, at one per A, S and C microcosm sets, were rinsed with sterile deionized water then disinfected with 99.9% (v/v) ethanol (Thermo Fisher Scientific, Loughbrough, U.K.) after each sampling to avoid microbial cross-contamination^69^. Future work will adopt a more stringent regime of cleaning all sampling equipment (core borers, spatulas) with bleach and/or portable sources of ultraviolet light to further minimize DNA cross contamination^59^. The samples were transported to the laboratory in a cold storage box containing ice packs and stored at -20 °C until processed. Collected samples were used to profile soil bacterial 16S rRNA community or metabarcode by next-generation sequencing (NGS), soil elemental composition by portable X-ray fluorescent (pXRF) spectrometry, and soil pH determination. Metabarcoding was made only for the top 0–30 cm soil layers of the A, S and C microcosms and the first 90 days of study since the *Mus musculus* had reached skeletonization after ~ 90 days.

### Temperature, accumulated degree days (ADD) and weather

Soil temperature and moisture were monitored in situ and continuously using R-C 4 H Temperature and Humidity data-loggers (Elitech Ltd., England). The following protocols were then performed as described by Bisker et al. (2021)^13^: environmental monitoring for daily atmospheric temperatures and UV index for Middlesbrough (North Yorkshire, U.K.); soil humidity; accumulated degree-days (ADD) and cumulative precipitation; soil mineral composition; soil microbial DNA extraction, polymerase chain reaction and next-generation sequencing; data and statistical analyses.

### Total body scoring (TBS)

Three mice were buried individually close to the front sides of three transparent PVC pipes and their stages of subsurface decomposition scored visually^70^ (TBS) according to the method suggested by Megyesi, Nawrocki, and Haskell (2005)^14^ and amended by Adlam and Simmons (2007)^15^. Specifically, Megyesi, Nawrocki, and Haskell (2005)^14^ assessed decomposition by scoring decomposing remains from 3 to 34. The head, trunk and limbs of a human cadaver are allocated a “score” each according to their level of decomposition on the corresponding table. The sum of the scores gives a total body score (TBS) inferring time since death. Adlam and Simmons (2007)^15^ used rabbits as human proxies and suggested a modified approach where physical changes determined the stages decomposition. Therefore, pictures of the mice decomposing aboveground and in the subsurface inside the transparent pipes were taken regularly (Supplementary Figure S7) for visual assessments to facilitate TBS. Together with soil pH monitoring, this would permit the assessment of the decomposition stage and detection of the moment of skin rupture and nutrient release into the soil. *Mus musculus* is a small human surrogate that, compared to a pig, allows for faster decomposition and an increase in environmental control and sample size. Mice and humans have similar skin permeability and share analogous biochemical, cellular, and physiological pathways^10^.

### Portable XRF analysis (pXRF)

The mineral composition of soil samples was assessed at each sampling time using a portable X-Ray fluorescence (pXRF) analyser, Thermo NitonTM XL3t 950 GOLDD + series Analyser (Niton UK Limited, Winchester, U.K.). Soil samples were dried overnight at 105 °C, ground up with a mortar and pestle, sieved and homogenized. Separate XRF cups (31 mm) were filled with approximately 8 g of soil from each individual treatment or control and covered with 5 μm polypropylene film before scanning for 30 s at the low beam and 60 s at the light beam using mining mode. Blank and standard samples were run after every 10 samples analyzed. The overall number is the value of all detected elements combined, which is essential to show how much of the inorganic elemental soil fraction has been analyzed across all the study soils.

### DNA extraction

DNA was extracted from each soil sample (500 mg) from specific intervals of the 1-year study using FastDNA™ Spin Kit for Soil™ (MP Biomedicals, U.K.) according to the manufacturer’s instructions and stored at ­20 °C until required. The concentration and purity (A_260_/A_280_) of each DNA extract was determined by UV spectrophotometry (Synergy-HT microplate reader; Biotek, Bedfordshire, UK) where a ratio of 1.8-2.0 was equivalent to high quality and high purity DNA^71,72^.

### Next-generation sequencing (NGS)

Due to the 3-month onset of *M. musculus* skeletonization, only the day 1 to day 90 soil samples were sequenced on the on Illumina MiSeqTM platform by NU-Omics (Northumbria University, Newcastle Upon Tyne, UK) using the bacterial V4 region 16S rRNA gene set of primers 515f (5’-GTGCCAGCMGCCGCGGTAA-3’) and 806r (5’-GGACTACHVGGGTWTCTAAT-3’)^73–75^. Preparation and sequencing of 16 S rRNA gene libraries were as described by Kozich et al. (2013)^73^.

### Data and statistical analysis

pXRF readings were performed separately for each individual replicate with Microsoft Office Excel^™^ Professional Plus 2016, and used subsequently to calculate the averages for each set of triplicate readings and plot graphs.

The raw reads resulting from next-generation sequencing were analysed using the Quantitative Insights Into Microbial Ecology version 2 (QIIME2) software suite version 2018.8^76–78^. Sequences were subsequently denoised, reads rarified to 4 000, and Amplicon sequence variants (ASVs) produced by the Divisive Amplicon Denoising Algorithm 2 (DADA2). UCHIME was used to quality check and filter the FASTA formatted sequences for chimeras before assigning taxonomy using RDP14 reference database^79^. ASV tables and metadata were also analysed using R version 4.2.1. Samples were faceted to rarefy based on sequencing depth and ASV totaling less than 0.01% of the overall data set were removed, relative abundance data was compiled. Stacked bar charts showing most abundant phyla and genera were created using ggplot2^80^. Reshape^81^ and variation between groups were determined using MaAsLin2 tool available within the Galaxy environment^82^. NMDS was created using vegan version 2.6-2 and ggplot2 packages and Bray-Curtis distance matrixes. Significant effects were measured using ANOSIM. Alpha diversity was measured using Shannon-diversity and Simpson index while Kruskal-Wallis test was used to determine significant differences in alpha-diversity between groups.

All data were evaluated by a two-way ANOVA for time and decomposition aboveground vs. in the subsurface with replication followed by Tukey HSD and Bonferroni to explore the interactions using IBM SPSS Statistics version 25 (IBM Corporation, New York, USA).

## Electronic supplementary material

Below is the link to the electronic supplementary material.

Supplementary Material 1

## Data Availability

The data generated in this article can be found in: BioProject https://www.ncbi.nlm.nih.gov/bioproject/PRJNA1111578; BioSamples https://trace.ncbi.nlm.nih.gov/Traces/study/?acc=SRP507637; and Bisker et al_Supplementary Material_Part 2.
