# Supplementary material for: Comparative soil bacterial metabarcoding after aboveground vs. subsurface decomposition of Mus musculus
Source: Sci Rep. 2024 Dec 28;14:31179. doi: 10.1038/s41598-024-82437-0 (PMC11682113; doi:10.1038/s41598-024-82437-0)
Supplement: Supplementary file 1 — Supplementary Material 1 [file 41598_2024_82437_MOESM1_ESM.docx]

**Supplementary Material_Part 2**

**Supplementary Figure S1.** Phylum-level bacterial resolution bar-plot of the control (C), subsurface (S) and aboveground (A) decomposing-mice associated soils at 30 cm deep on days 30, 60 and 90.

B

A

Deposition diversity (P=0.093372) Temporal diversity (P=0.133335)

D

C

Deposition richness (P=0.001168) Temporal richness (P=0.213514)

**Supplementary Figure S2.** Shannon diversity (A, B) and richness (C, D) in response to mouse deposition aboveground vs. subsuface and no-mouse control, and sampling time.

**Supplementary Figure S3.** Average (n=3) soil pH of the subsurface (⚫), aboveground (⚫) and control (●) soils during a 360-day study. Error bars are SEM.

**Supplementary Figure S4.** Atmospheric temperature, UV index (A); Accumulated degree-days (ADD) (B) calculated for the subsurface (●), aboveground (●) and subsurface decomposition in clear pipes (●) during the 360-day study.


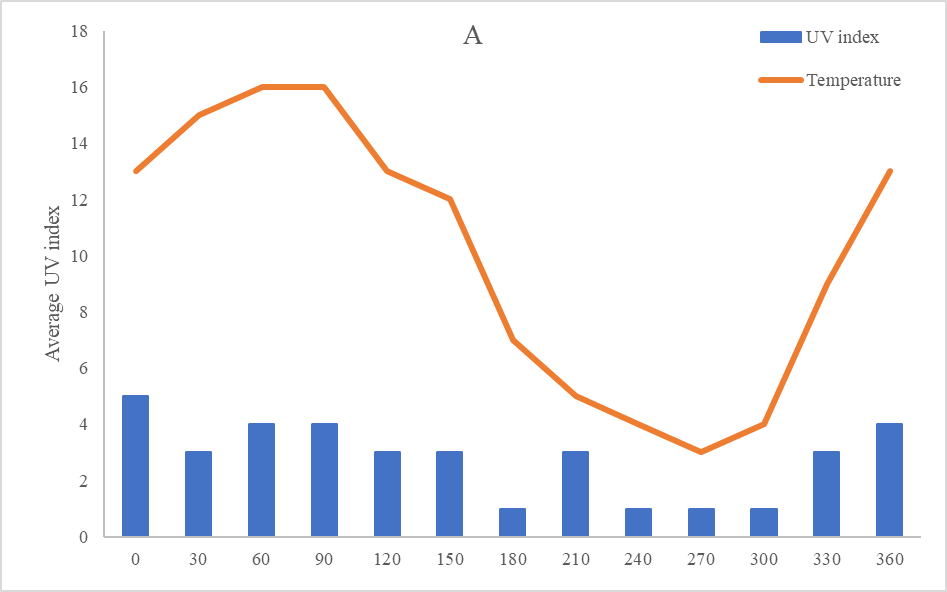

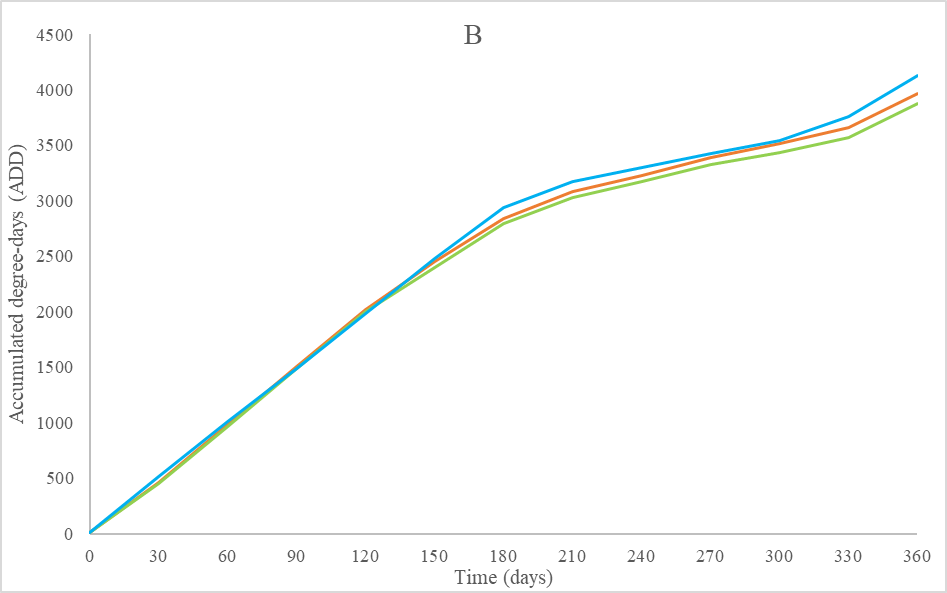

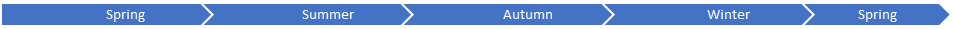


**Supplementary Figure S5.** Average (n = 3) *M. musculus* soil composition in iron (Fe; ◼), potassium (K; ◼), calcium (Ca; ◼), phosphorus (P; ◼), sulphur (S; ◼) and magnesium (Mg; ◼) for (A) aboveground sampled on days 0 and 360 only, (B) aboveground sampled regularly, (C) subsurface sampled on days 0 and 360 only and (D) subsurface sampled regularly.


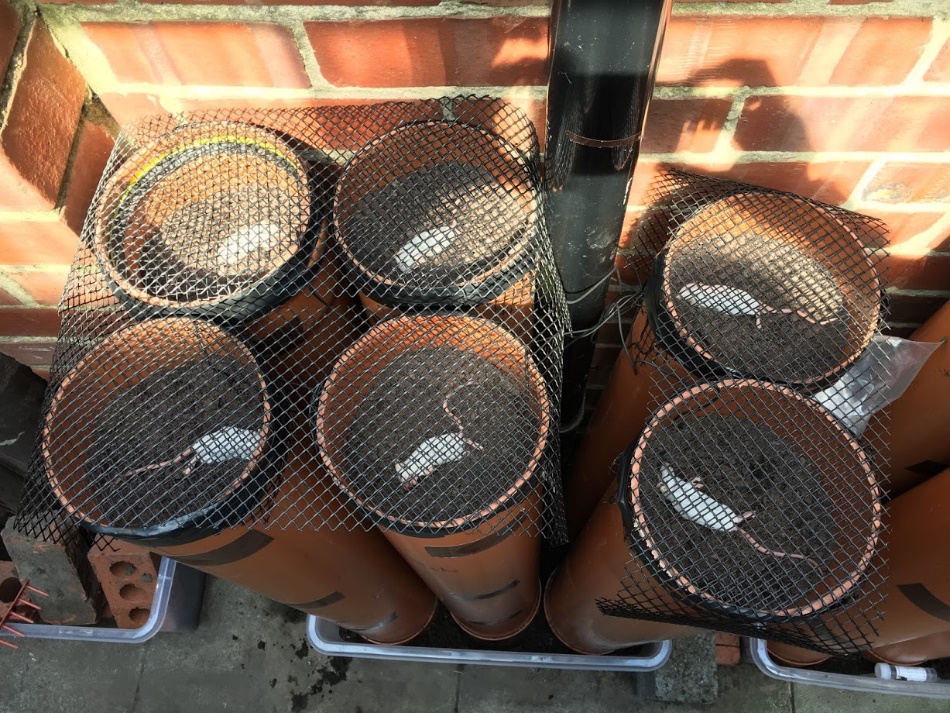


A

A2


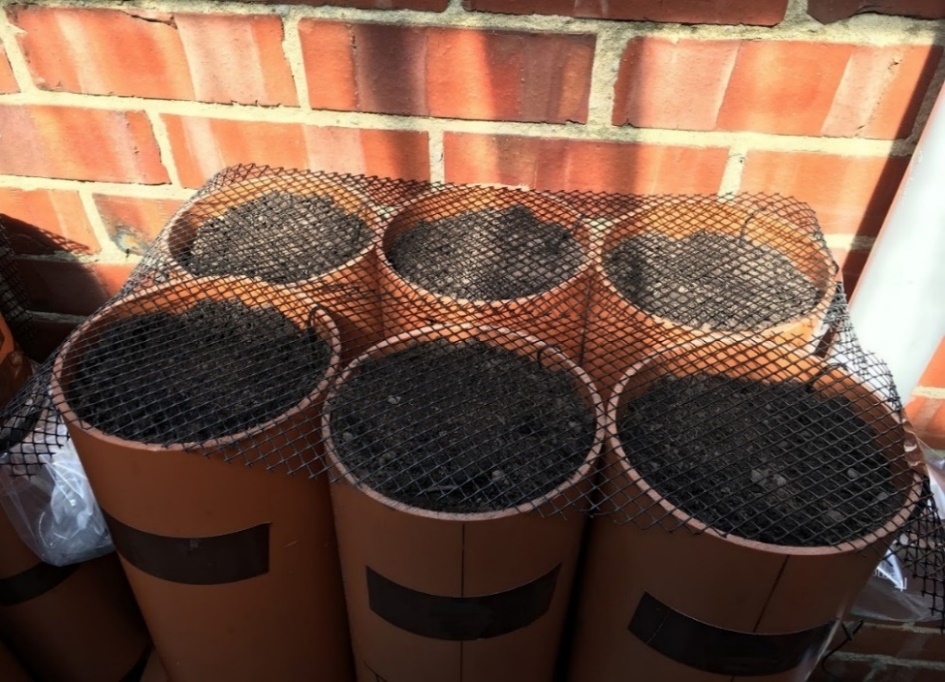


S

S2


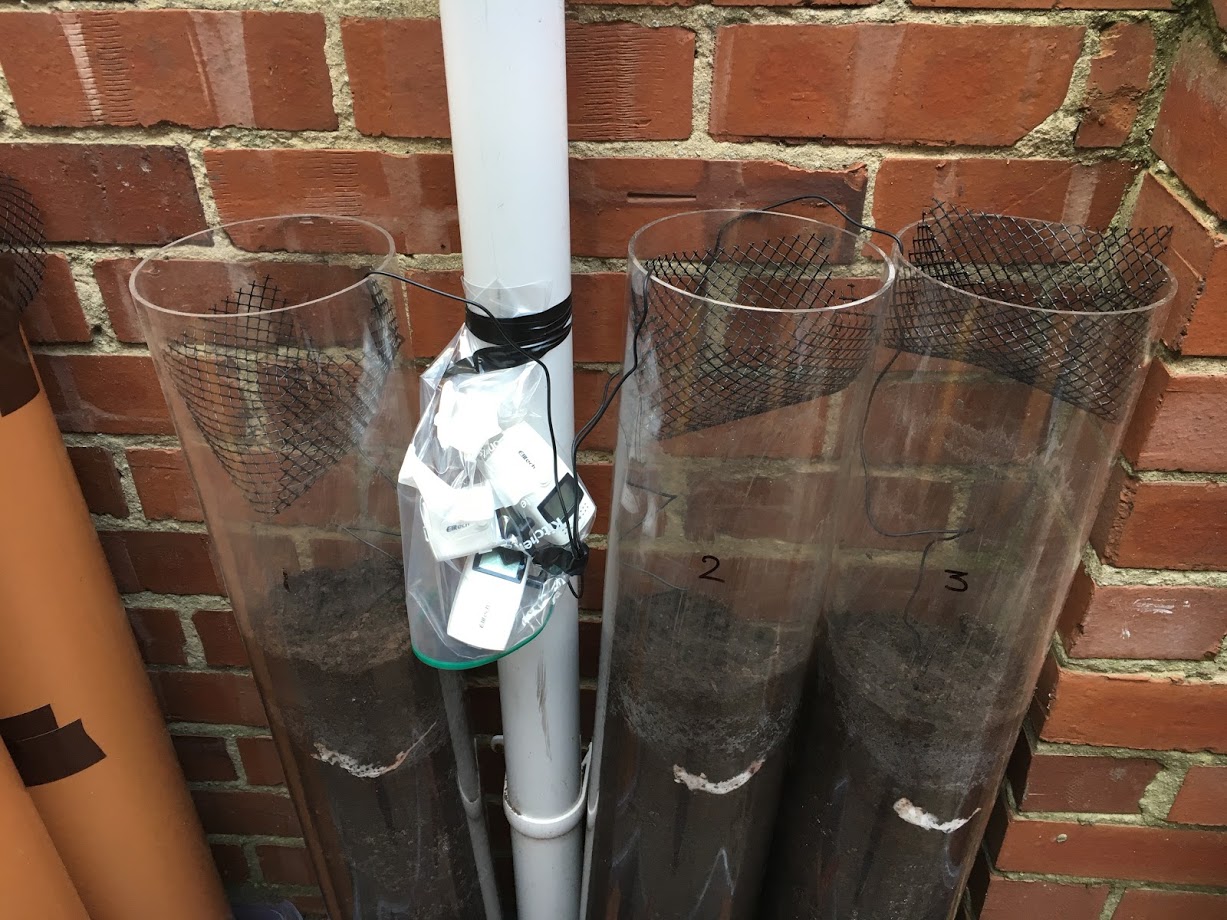


TBS

**Supplementary Figure S6.** Images of the *Mus musculus* decomposition microcosms: Aboveground sampled regularly (A) and at days 0 and 360 only (A2); Subsurface sampled regularly (S) and at days 0 and 360 only (S2); and in transparent U-PVC pipes for total body score (TBS) measurements.

**Week 1 10 days 30 days 70 days 90 days**


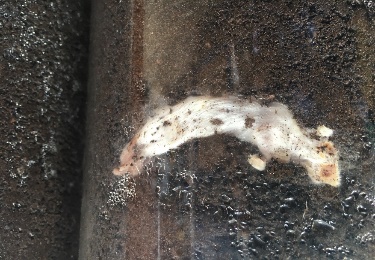

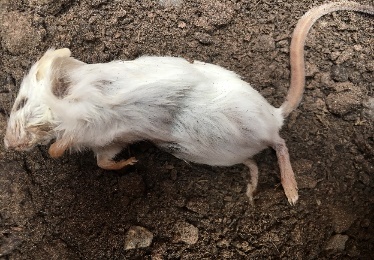

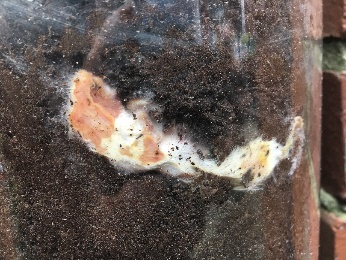

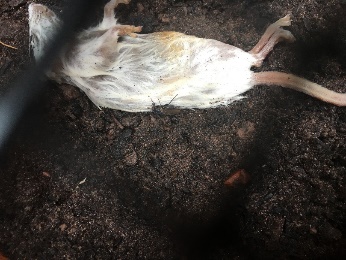

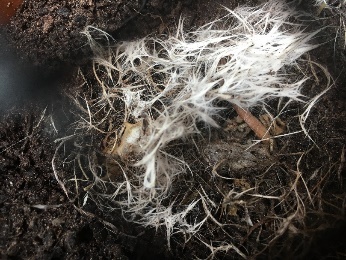

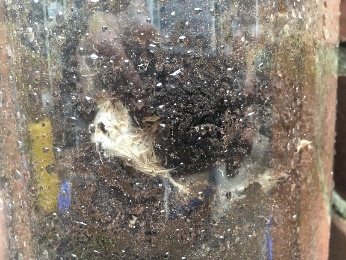

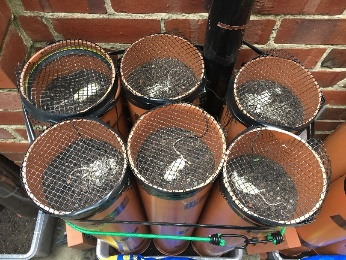

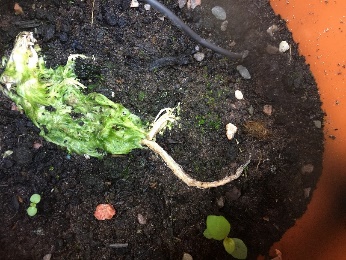

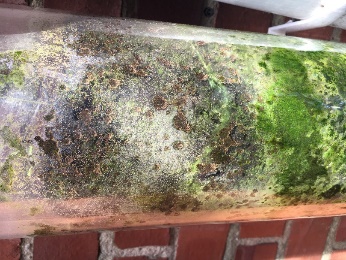

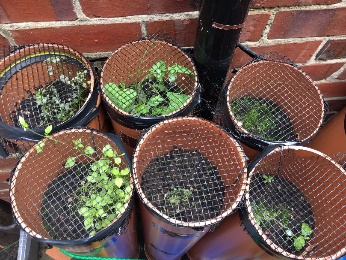

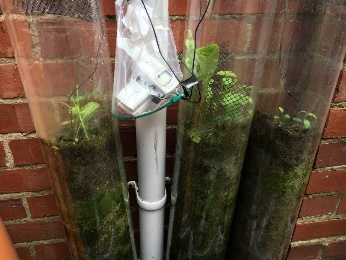


**TBS**


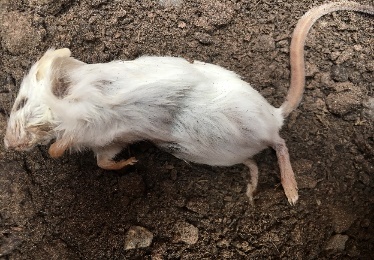


**A**

**Supplementary Figure S7.** Images of the decomposition microcosms with *Mus musculus* in transparent U-PVC pipes for total body score (TBS) measurements and aboveground (A).
